# Estimating the potential impact of the Australian government’s reformulation targets on household sugar purchases

**DOI:** 10.1186/s12966-021-01208-6

**Published:** 2021-10-28

**Authors:** Daisy H. Coyle, Maria Shahid, Elizabeth K. Dunford, Jimmy Chun Yu Louie, Kathy Trieu, Matti Marklund, Bruce Neal, Jason H. Y. Wu

**Affiliations:** 1 Faculty of Medicine, The George Institute for Global Health, UNSW Sydney, Level 5, 1 King St, Newtown, Australia; 2 Department of Nutrition, The University of North Carolina at Chapel Hill, Chapel Hill, USA; 3 School of Biological Sciences, Faculty of Science, The University of Hong Kong, Pokfulam, Hong Kong, SAR China; 4 Department of Epidemiology, Johns Hopkins Bloomberg School of Public Health, Baltimore, MD USA

## Abstract

**Background:**

Countries around the world are putting in place sugar reformulation targets for packaged foods to reduce excess sugar consumption. The Australian government released its voluntary sugar reformulation targets for nine food categories in 2020. We estimated the potential impact of these targets on household sugar purchases and examined differences by income. For comparison, we also modelled the potential impact of the UK sugar reduction targets on per capita sugar purchases as the UK has one of the most comprehensive sugar reduction strategies in the world.

**Methods:**

Grocery purchase data from a nationally representative consumer panel (*n*=7,188) in Australia was linked with a large database (FoodSwitch) with product-specific sugar content information for packaged foods (*n*=25,261); both datasets were collected in 2018. Potential reductions in per capita sugar purchases were calculated overall and by food category. Differences in sugar reduction across income level were assessed by analysis of variance.

**Results:**

In 2018, the total sugar acquired from packaged food and beverage purchases consumed at-home was 56.1 g/day per capita. Australia’s voluntary reformulation targets for sugar covered 2,471/25,261 (9.8%) unique products in the FoodSwitch dataset. Under the scenario that all food companies adhered to the voluntary targets, sugar purchases were estimated to be reduced by 0.9 g/day per capita, which represents a 1.5% reduction in sugar purchased from packaged foods. However, if Australia adopted the UK targets, over twice as many products would be covered (*n*=4,667), and this would result in a more than four times greater reduction in sugar purchases (4.1 g/day per capita). It was also estimated that if all food companies complied with Australia’s voluntary sugar targets, reductions to sugar would be slightly greater in low-income households compared with high-income households by 0.3 g/day (95%CI 0.2 - 0.4 g/day, *p*<0.001).

**Conclusions:**

Sugar-reduction policies have the potential to substantially reduce population sugar consumption and may help to reduce health inequalities related to excess sugar consumption. However, the current reformulation targets in Australia are estimated to achieve only a small reduction to sugar intakes, particularly in comparison to the UK’s sugar reduction program.

**Supplementary Information:**

The online version contains supplementary material available at 10.1186/s12966-021-01208-6.

## Introduction

Excess sugar intake is associated with adverse health outcomes including unhealthy weight gain [1, 2], dental caries [3] and type 2 diabetes [4, 5]. The World Health Organization (WHO) recognizes the importance of reducing population sugar consumption and recommends that adults and children reduce their daily intake of ‘free’ sugar (which includes added sugars as well as naturally occurring sugars in honey, syrup and fruit juice) to less than 10% of total energy [6]. In Australia, approximately half of the adult population (47% of men and 44% of women) exceeds this recommendation [7]. Intakes of free sugar are particularly high in young adults (56% and 59% of men and women aged 18-30 years consume ≥10% energy from free sugar, respectively) and in individuals with a low socioeconomic (SES) status (~50% exceed the recommended level of intake) [7].

Sugars added to processed foods is a major dietary source of free sugars globally [8–10]. In an attempt to achieve population-wide reductions in free sugar intake, a growing number of governments have adopted policies to reduce the amount of sugar added to processed foods [11, 12]. For instance, the UK has one of the most comprehensive sugar reduction strategies in the world, which aims to reduce the amount of total sugar in targeted food and beverage products by 20% through a multi-component approach that includes reformulation targets and portion size guidelines [12]. Policies targeting the reduction of sugar in the food system have tremendous potential to improve population health – for example, a modelling study has shown that if the UK sugar reduction program was achieved in its entirety, it could reduce sugar consumption in adults by 5%, preventing up to 154,000 cases of diabetes over 10 years and generating an estimated ~50,000 quality adjusted life years [13].

In Australia, a large proportion of free sugars in the diet comes from energy-dense, nutrient-poor discretionary products, such as sugar-sweetened beverages and cakes [8, 14]. To reduce population sugar intake, the Australian Federal government has released a series of voluntary reformulation targets as part of the Healthy Food Partnership initiative [15, 16]. The Healthy Food Partnership was established in 2015 and is a public-private partnership between government, the public health sector and the food industry that aims to improve the dietary habits of the Australian population [16]. The voluntary reformulation targets for total sugar were released in 2021 across nine packaged food and beverage categories [17].

The primary aim of this study was to estimate the potential impact of the Healthy Food Partnership sugar reformulation targets on the amount of sugar brought into the home from packaged food purchases. We examined the potential impact overall and for households with different income levels, since prior literature suggests lower socio-economic status groups consume higher intakes of sugar [8, 18–20]. We also modelled reductions across food categories to estimate where the greatest sugar reductions are likely to be achieved. In addition, we modelled the amount of sugar reduction that could be achieved if Australia had adopted the UK targets, in order to gain insight into how the Australian targets compare against a more comprehensive sugar reduction program.

## Method

This project was approved by the University of New South Wales Human Research Ethics Committee (approval number HC180965).

### Study design and population

This study used data from the NielsenIQ Homescan Consumer Panel [21], a commercial dataset that contains information on food and beverage purchases made by Australian households. Data over a one-year period (January– December 2018) was used for the current analysis, which contained information from approximately 10,000 households who recorded all food and beverages purchased and brought into the home throughout the year. Participating households are recruited to ensure the panel is representative of the demographic composition and geographic location of Australian households [22]. The dataset contains information on the sociodemographic characteristics of the households, including ethnicity and education level of the main shopper in the household, household income, and age and sex of all household members. This dataset is frequently used by researchers to examine food and beverage purchasing habits as a proxy for consumption [22–25].

Participating households are provided with a handheld electronic scanner and are instructed to scan the barcodes of all packaged food and beverage items purchased from retail outlets including supermarkets and convenience stores. Data are not collected on foods and beverages purchased and consumed outside of the home, for example from restaurants and take-away outlets. To best capture usual shopping purchases made throughout the year and to account for products that are stored and not consumed immediately, we analyzed year-level purchases by summing all purchases made during the 2018 calendar year.

### Household exclusion criteria

Using NielsenIQ quality standard criteria, we excluded households if they were not on the panel for the entire 52-week time frame, if they did not record any purchases (at least one barcode per week) for at least 50% of the weeks, if they were missing any demographic information or if they did not meet minimum spend criteria (≥$5 on average for each week for all purchases). To further exclude households that may have under-reported purchases, households with the lowest annual food and beverage expenditure (<2.5^th^ percentile defined separately for single-member households and multi-member households) were also excluded [22].

### Food and beverage nutrient data

Each barcoded product purchased by Homescan households was linked to its corresponding nutrition information panel (NIP) to obtain the total sugar content. Information from the NIP was obtained from the 2018 FoodSwitch annual database [26–28], which contains nutrition information for packaged food and beverage products available for sale from five large supermarket retailers in Australia (Woolworths, Coles, Aldi, IGA and Harris Farm) who together make up about 80% of the market share of packaged grocery products in Australia [29]. This information was collected by trained personnel during in-store collections in the months of August to November in 2018 (no crowd-sourced data was used in this study). Using the categorization system developed by the Global Food Monitoring Group, each food and beverage product in the FoodSwitch database was classified into a hierarchical category tree to allow for comparison of nutritionally similar foods [22, 27, 30, 31]. This classifies products into food groups (e.g. non-alcoholic beverages), categories (e.g. fruit and vegetable juices), subcategories (e.g. fruit juice) and leaf categories (e.g. apple juice).

A total of 22,738 products were directly linked to their nutritional information via their unique barcodes, which accounted for 85% of the quantity of product units purchased by the households. To further improve the coverage of products purchased by households, we applied additional matching steps using previously described methods [22, 32]. This included linking products by their (i) product name and (ii) product name after removing nutritionally irrelevant descriptors such as size and shape information. After applying these additional matching steps, the number of matched products increased to 25,261, representing 88% of the total quantity of products purchased by households in 2018.

### Modelled scenarios

#### Australian government’s sugar reformulation targets

Australia’s sugar reformulation targets contain both maximum and proportional sugar targets across nine food and beverage categories. The food categories (and their target levels) include: breakfast cereals with fruit (22.5 g/100 g and at least a 20% reduction for products containing more than 28 g sugar/100 g); breakfast cereals without fruit (20 g/100 g and at least a 20% reduction for products containing more than 25 g sugar/100 g); flavoured milk - mammalian (9 g/100 mL); flavoured milk - dairy alternatives (5 g/100 mL); muesli and snack bars (25 g/100 g and at least a 15% reduction for products containing more than 28.5 g sugar/100 g), flavoured water (5 g/100 mL), flavoured mineral water, soda water and iced tea, carbonated soft drinks and energy drinks (a 10% reduction for products containing more than 10g sugar/100 mL), fruit drinks (9.5 g/100 mL) and sweetened yoghurt (mammalian) (12.5 g/100 g). Further details about each of the targeted food categories is provided in Supplementary Table 1.

The first step in our modelling analyses was to map the nine categories to the FoodSwitch dataset to identify which foods and beverages fall under each of these targeted food categories. Using the Australian government’s Healthy Food Partnership reformulation food category definitions [17], a researcher within the study team manually linked each of the targeted food categories to the relevant leaf categories (the finest category level) in FoodSwitch. For example, a number of leaf categories were mapped to the ‘Fruit Drink’ sugar target including ‘apple juice’, ‘apple-mango juice’ and ‘pineapple juice’. Targets were linked to leaf categories to reduce the risk of irrelevant products being mapped to the targets.

We modelled the potential reductions to Australian household sugar purchases by comparing the current amount of sugar purchased from packaged foods and beverages to a ‘best-case’ scenario whereby all products were successfully reformulated to meet the targets [17]. For food categories with a maximum target, foods and beverages with a sugar content at or below the target level were assumed to retain their existing sugar content, whereas, foods and beverages with a sugar content above the target level were hypothetically reformulated so that the sugar content was equal to the category-specific sugar target. For example, in the sweetened yoghurt category where the target is 12.5 g/100 g, if a sweetened yoghurt had a sugar level of 10 g/100 g, then we assumed its sugar content would remain the same. Conversely, if a sweetened yoghurt had a current sugar content of 15 g/100 g, then we assumed it would be reformulated to 12.5 g/100 g. For food and beverage categories with a proportional reduction target, a 10% reduction in the sugar content was applied to products that exceeded the category-specific threshold. For example, in the carbonated soft drinks and energy drinks where a 10% reduction target applies to products containing more than 10 g of sugar per 100 mL, if a product had a sugar content of 9 g/100 mL then we assumed the sugar content would not change. However, if a product had a sugar content of 12 g/100 mL then we assumed it would be reformulated to 10.8 g/100 mL. As it is unclear whether product reformulation can lead to changes in dietary behaviour, we assumed that purchasing habits would not be affected by reformulation (i.e. baseline purchases would remain the same post reformulation).

#### UK government sugar reduction targets

We also modelled the potential impact of the UK sugar reduction program on household sugar purchases [12]. This program aims to reduce the total sugar content of targeted products by 20% through three key strategies: reformulation to lower the sugar content of products, portion size guidelines to reduce the portion size of single serve foods, and shifting consumer purchasing towards lower/no added sugar products (Supplementary Table 2). To model potential reductions, we applied a 20% reduction in the 2018 sugar content of all targeted foods and beverages. For example, if a yoghurt had a sugar content of 10 g/100 g, then we assumed the sugar content would be reduced to 8 g/100 g. This was applied across each relevant product purchased by Australian households in 2018 to ensure reductions were weighted according to sales. Following the UK guidelines, a small allowance for naturally occurring sugars (lactose) for products in the yoghurt category was made by removing 3.8 g of sugar per 100 g from the total sugar content before applying the 20% reduction.

### Statistical analysis

We first calculated the number of unique products targeted by the Australian and UK sugar reduction programs, as well as the proportion (%) of these products purchased by Australian households in 2018 that already met the Australian and UK targets. We then calculated current sugar purchases per capita from targeted food categories i.e. the amount of sugar in grams (as indicated on pack) purchased daily per person, by dividing total household sugar purchases per year, by 365 days, and by the number of individuals within the household. We then calculated sugar purchases per capita with the sugar reduction targets applied to assess how much sugar Australians would purchase after full adoption of the Australian and UK sugar reduction programs. We used the difference in sugar purchases before and after application of the sugar targets to calculate the change in mean sugar purchases per capita.

We also explored changes to mean sugar purchases per capita across household income level. The Organisation for Economic Co-operation and Development (OECD)-modified equivalized scales were applied to calculate gross equivalized household income [33, 34]. Three income groups were generated with an approximately equal number of households in each group: low-income households (<AUD$28,750 per year); middle-income households (AUD$28,750 - $55,000 per year) and high-income households (>AUD$55,000 per year). Reductions in per capita sugar purchases were compared across groups using 1-factor ANOVA with post-hoc pairwise tests.

To ensure the NielsenIQ Homescan panel is representative of socio-economic, demographic and geographic composition of the Australian population, NielsenIQ uses census data [35] to generate sample weights to allow findings based on the panel to be projected up to the Australian population in 2018. All of the results presented in this paper use the sample weights provided by NielsenIQ and are representative of the Australian population [35]. All statistical analyses were performed using Stata V.16.0 (StataCorp). A two-sided p-value of <0.05 was considered statistically significant.

To explore the potential influence of under-reporting, a sensitivity analysis was conducted whereby households whose annual food and beverage expenditure were ≤5th percentile were excluded, with the percentile value defined separately for single and multi-member households.

## Results

### Characteristics of the NielsenIQ Homescan Panel

In 2018, the Homescan panel included 11,056 households. Of these, 3,868 were excluded for not meeting eligibility criteria, leaving 7,188 households for the current analysis (Supplementary Figure 1). The main household shopper tended to be female (68.4%) and over the age of 40 (88.4%). Most households consisted of one or two persons (56.8%), which is consistent with Australian census data from 2016 (58%) [35]. In terms of household composition, the most common was families with children aged 11-17 years old (41.4%), followed by older singles and couples over the age of 45 (23.6%). The mean equivalized annual household income across all households was $46,987 AUD. The education level of the main household shopper was mainly high school/trade/diploma (41.5%), followed by bachelor’s degree or higher (22.3%), and less than high school (20.5%). A description of household characteristics is provided in Supplementary Table 3.

### Overall impact of the Australian reformulation program on per capita sugar purchases

Across the 25,261 unique products that matched across the Nielsen and FoodSwitch databases, only 9.8% were in product categories eligible for a sugar reformulation target (n = 2,471), which represented 9.5% of all products purchased by Australian households in 2018. Just over half (52.4%) of these targeted products already met the sugar targets. In terms of beverage categories, this ranged from as low as 26.3% for fruit drinks up to 83.3% for flavoured waters. For the food categories, this ranged from 49.6% for muesli and snack bars to 69.8% for sweetened yoghurt (Table 1).

**Table 1:** Modelled impact of the Australian sugar reformulation targets on Australian household purchases of sugar

Food category^a^	Sub-category	Number of unique products subject to reformulation	Products already meeting target (%)	Mean sugar purchases (g/d per capita)^b,c^		
				Current	Reformulated to meet targets^d^	Difference (Current – targets applied)
All categories combined		2471	52.4	10.22	9.36	0.87
Breakfast Cereals	Breakfast cereals with fruit	226	65.0	0.85	0.79	0.06
	Breakfast cereals without fruit	222	58.9	0.94	0.83	0.11
Flavoured milk	Flavoured milk: Mammalian	177	42.5	0.71	0.65	0.05
	Flavoured milk: Dairy alternatives	29	59.4	0.02	0.02	0.00
Muesli and snack bars	Muesli and snack bars	310	49.6	0.45	0.41	0.03
Non-alcoholic beverages	Flavoured water, mineral water, soda water and iced tea	326	83.3	0.22	0.20	0.01
	Carbonated soft drinks and energy drinks	576	29.5	5.12	4.69	0.44
	Fruit drinks	130	26.3	0.81	0.74	0.07
Sweetened yoghurt	Sweetened yoghurt: Mammalian	475	69.8	1.10	1.01	0.09

^a^Food categories and sub-categories listed are those that are targeted by the Australian government’s sugar reformulation program

^b^Results are sales-weighted and are projected to the Australian population using sample weights provided by NielsenIQ

^c^Standard error (SE) for mean sugar purchases (g/d per capita) not displayed as SE ≤0.01 for each mean value

^d^For food categories with a maximal target, foods and beverages with a sugar content at or below the target-level retained their existing sugar content, whereas, foods and beverages. For food categories with a proportional reduction target, a 10% sugar reduction was applied to products that met the criteria

In 2018, the total sugar (mean±SE) purchased from these targeted food categories at baseline was 10.2±0.0 g/day per capita, representing 18.2% of all sugar purchased from packaged foods and beverages in 2018 (56.1±0.0 g/day per capita). This was predominately attributed to purchases of carbonated soft drinks and energy drinks (5.1 g/d per capita, 50.1% of all sugar purchased across targeted food categories), sweetened yoghurt (1.1 g/d per capita, 10.8%) and breakfast cereals without fruit (0.9 g/d per capita, 9.2%) (Table 1). It was estimated that if food companies were to reformulate all existing products to meet Australia’s sugar targets, this would reduce baseline mean sugar purchases by 0.9 g/day per capita, representing an 8.5% reduction in sugar across targeted food categories and a 1.5% reduction in sugar purchased from all packaged foods and beverages. Across the nine food categories targeted, the mean reductions were projected to be largest for carbonated soft drinks and energy drinks (0.4 g/d per capita, 50.3% of all projected reductions), breakfast cereals without fruit (0.1 g/d per capita, 13.1%) and sweetened yoghurt (0.1 g/d per capita, 10.1%) (Table 1).

Across the targeted food categories, per capita sugar purchases were marginally higher for low-income households compared to high-income households (2.5g/day, 95%CI: 1.6 - 3.5g/day, *P*<0.001) (Table 2). If all food companies met the targets in full, this would result in a marginally greater absolute reduction in per capita sugar purchases for low-income households compared with high-income households (mean difference 0.3 g/day, 95%CI 0.2 - 0.4 g/day, *P*<0.001).

**Table 2:** Modelled impact of the Australian sugar reformulation targets on changes to sugar purchases (g/d per capita), by income level

	Mean sugar purchases across targeted food categories (g/d per capita)^b,c^		
Income level^a^	Current	Reformulated to meet targets^d^	Mean difference (current – targets applied)
Low	11.64	10.63	1.01
Middle	10.30	9.42	0.88
High	9.17	8.41	0.75

^a^The OECD-modified equivalence scale was applied to calculate equivalised household income (adjusting for household size and age of household members). Three income groups (low, middle and high) were then generated by splitting households into three groups of approximately equal numbers. The three household income groups (low: <$28,750 per year, middle: $28,750 - $55,000 per year and high: >$55,000 per year) had mean incomes that were comparable to equivalised incomes for the Australian population in 2016 (low income: ≤30th percentile ≤$33,020; middle-income: 30–60th percentile: $33,021–51,324, high-income: >60th percentile ≥$51,325)

^b^SE for sugar purchases (g/day per capita) not displayed as SE ≤0.01 for each mean value

^c^Results are sales weighted and are projected to the Australian population using sample weights provided by NielsenIQ

^d^Foods with per 100g sugar values at or below the target retained their sugar content, whereas foods with per 100g sugar values above the target had the sugar content replaced with the sugar target

### Potential impact if Australia adopted the UK sugar reduction targets

The UK sugar reduction targets cover approximately twice as many products as the Australian targets (4,667 versus 2,471 unique foods and beverages). The total sugar (mean±SE) acquired from the UK targeted food categories at baseline was 20.9±0.0 g/day per capita, representing 37.3% of all sugar purchased from packaged foods and beverages in 2018. This was predominately attributed to purchases of ice cream, lollies and sorbets (4.8 g/d per capita, 22.7% of all sugar purchased across targeted food categories), chocolate confectionery (4.4 g/d per capita, 20.9%) and biscuits/cookies (2.9 g/d per capita, 13.7%) (Table 3).

**Table 3:** Modelled impact of the UK sugar reduction targets on Australian household purchases of sugar

Food category^a^	Number of unique products subject to sugar reduction	Mean sugar purchases (g/d per capita)^b,c^		
		Current	Sugar levels reduced by 20%	Difference (Current – targets applied)
Overall	4667	20.94	16.87	4.07
Breakfast cereals	524	1.89	1.51	0.38
Biscuits/cookies	607	2.87	2.29	0.57
Cakes	283	0.87	0.70	0.17
Chocolate confectionery	890	4.37	3.49	0.87
Ice cream, lollies and sorbets	560	4.76	3.81	0.95
Morning goods	106	0.35	0.28	0.07
Puddings	460	1.23	0.99	0.25
Sweet confectionery	483	2.11	1.69	0.42
Yoghurts	456	1.09	0.94	0.15
Sweet spreads and sauces	298	1.38	1.14	0.23

^a^Food categories and sub-categories listed are those that are targeted by the UK government’s sugar reduction program

^b^Results are sales-weighted and are projected to the Australian population using sample weights provided by NielsenIQ

^c^Standard error (SE) for mean sugar purchases (g/d per capita) not displayed as SE ≤0.01 for each mean value

It was estimated that if all food and beverage companies were to reformulate their products to meet the UK sugar reduction targets, the impact on sugar purchases would be more than four-times greater compared with the current Australian targets, resulting in a mean reduction of 4.1 g/day per capita (Table 3). This is equivalent to a 7.3% reduction in total sugar from all packaged food and beverage purchases at baseline. A large proportion of the per capita reduction from the UK targets came from food categories not covered by the Australian targets including ice cream, lollies and sorbets (1.0 g/day), chocolate confectionery (0.9 g/day) and biscuits/cookies (0.6 g/day) (Table 3).

It was estimated that low-income households had significantly higher per capita sugar purchases from the food categories targeted by the UK targets compared to high-income households (by 5.8g/day, 95%CI: 4.7 – 7.0g/day, P<0.001) (Supplementary Table 4). If all food companies met the targets in full, this would result in a marginally greater absolute reduction in per capita sugar purchases for low-income households compared with high-income households (mean difference 1.1 g/day, 95%CI 0.9 - 1.4 g/day, *P*<0.001).

### Sensitivity analysis

Excluding households in the lowest 5th percentile for total annual spend for foods and beverages did not appreciably change sugar reduction estimates for either the Australian or UK target scenarios at 0.9 g/d per capita and 4.2 g/d per capita, respectively.

## Discussion

Our analyses indicate that Australia’s voluntary sugar reformulation targets will likely have only a small impact on reducing sugar consumption across the Australian population. This is a concern considering excess sugar intakes can increase energy intakes and reduce overall diet quality, which may lead to weigh gain and increased risk of non-communicable disease [1, 5, 6]. With almost half of the Australian population consuming free sugar in excess, the ongoing absence of credible mechanisms to address high sugar levels in packaged foods and beverages is a cause for concern.

Our analyses suggest two critical weaknesses of the current Australian sugar reduction program. First, the target levels for sugar reduction are too lenient, with more than a half of the products covered by the program already meeting the reformulation targets prior to implementation. This conflicts directly with the stated aim of the reformulation program specified by government at the outset, which indicated that the targets would be set at a level whereby only one-third of products would already be at or below target [15]. The second issue is that the coverage of the program is too narrow; missing high sugar food categories including ice cream, lollies and sorbets, chocolate confectionery and biscuits/cookies [8, 20, 36]. The government could resolve these issues by setting lower sugar target levels to ensure only one-third of products meet the targets at baseline (thereby requiring a greater proportion of products to be reformulated), targeting a broader range of sugary food categories, and by introducing a wider range of sugar reduction policies to complement reformulation. The impact of these omissions is objectively demonstrated by comparison against the UK program, which if applied in Australia would cover twice as many products and deliver more than four times the sugar reduction. The addition of portion size guidelines to Australia’s sugar reduction program, whereby hard-to-reformulate products can be provided in smaller size packs, may provide opportunities to generate further impact on population sugar consumption.

Several technical issues are likely to have hampered the Australian government in designing the current sugar reformulation program. Firstly, the targets were set using information about the sugar content in products from a nutrition composition database with limited market coverage (a total of ~40,000 products since it started in 2014) [37] compared with >20,000 products collected each year from FoodSwitch (the database used in this analysis) [38]. The government also elected not to use purchase data during the target setting process and therefore were unable to account for the different quantities of products purchased by Australian households [15]. Use of purchase data would have added a further level of rigour to the target setting process, particularly in food categories where the sugar content of market-leading products differs substantially from the category average. The government was aware of the limited impact of these targets as they commissioned modelling work during the target setting process to estimate the likely impact of the reformulation targets on population diets [15]. Similar to our estimates in this study, this modelling work estimated the under that scenario that all food companies complied with the targets, sugar intakes would be reduced by just 1.3 g/d capita [15]. The small estimated reductions highlight the need for the government to introduce a more multi-sectoral approach for setting nutrition policies in Australia. While this could involve some engagement with the food industry regarding potential policies, representatives from the food industry should not be involved in setting nutrition policies in accordance with best practice as recommended by the WHO [39].

There was ample opportunity for the government to revise the targets during the target setting stage to leverage the extensive work undertaken by the UK government [40]. In addition to delivering targets that could have achieved significant reductions in sugar intake, if Australia had adopted the UK program in Australia, this would also have saved considerable time since the UK targets have been available since 2017; a full four years before the release of the Australian targets [17]. Adapting the UK targets to the Australian context should be feasible given these countries have comparable food supplies in terms of types of food available for sale and their nutritional quality [40]. However, as the UK sugar reduction program is much more comprehensive, adopting these targets in Australia would require political will and it may be difficult to generate such strong support from policymakers.

While already small in magnitude, our estimate of the impact of the Australian sugar reduction program is certainly optimistic. Given this is a voluntary program with no sanction for companies that fail to meet the targets, it is very unlikely that the food industry will fully comply, particularly given the previous reformulation targets in Australia showed varying levels of uptake across food companies with some achieving full reformulation across their products and others achieving very little [41–43]. Strong political engagement, media pressure and regular monitoring of progress are key to the successful implementation of voluntary policies such as Australia’s sugar targets [43, 44]. There is, however, no evidence that these accountability measures will be used by the Australian government to help deliver upon these very modest targets [43].

Our modelling estimated the effects of the Australian sugar reduction targets on total sugar purchases from packaged foods but the estimated effect on free sugars is similarly inconsequential. Australian adults consume on average 60g of free sugar per day [15], the current reformulation program would reduce daily free sugar intake by only about 1.5%. Even full adoption of the UK targets in Australia would fail to reduce average Australian free sugar intake (currently about 17% of daily energy intake) to a level close to the WHO recommended maximum of 10% of daily energy intake [6]. To achieve needed reductions in population free sugar consumption, our findings suggest that multiple policies rather than just reformulation may be needed, particularly as reformulation can face technological limitations in regard to food safety and consumer acceptability of taste, appearance and texture [45] and may not be the most feasible approach for all foods and beverages. Previous research has identified five action areas for reducing population intakes of free sugar in Australia [44]. These include prioritising health in policy and trade agreements; introducing fiscal policies to support health and promote reformulation; improving food labelling and advertising regulations; strengthening Australian dietary guidelines and introducing measures to encourage healthier choices, such as increasing healthy food and beverage offerings at hospitals, universities and workplaces [44].

Our findings are consistent with prior studies indicating that individuals from more deprived socio-economic backgrounds are more likely to consume diets higher in sugar [8, 18–20]. Despite the relatively weak sugar reformulation policy in Australia, our analysis has demonstrated that the targets could lead to small, albeit statistically significant, differences in absolute sugar reduction between high and low income households, thereby demonstrating the potential for upstream policies, such as reformulation, to reduce socioeconomic inequalities in sugar intakes. This is evident through our finding that the UK sugar reduction targets, which are more comprehensive and stringent compared to Australia’s targets, holds greater promise to deliver more meaningful sugar reductions for low socio-economic households. While we have quantified the potential absolute differences in sugar reduction across households of different income levels, future studies should assess how Australia’s reformulation program could translate into other outcomes impacted by socio-economic status, such as non-communicable disease risk. Given the need to reduce socio-economic disparities in diet across the Australian population, future modelling studies should look to assess a range of different reformulation scenarios to determine which strategies are likely to have the greatest impact on reducing sugar intakes across the lowest socio-economic groups. Such research will be critical for informing future reformulation policies in Australia.

A major strength of this study was our use of contemporary purchase data from a representative sample of Australian households [46]. Moreover, we were able to match this data to an up-to-date packaged food database containing product-specific sugar content information collected from grocery retailers, which together represent about 80% of total market share in Australia [29]. Purchase data and sugar content levels were directly linked for each individual product using unique barcode and product name information. This not only minimised the risk of errors related to the sugar content, it also ensured our results accurately reflected both the purchasing habits of Australian households and the nutritional composition of the food supply. This information allowed us to forecast the potential impact of the Australian reformulation program on sugar purchases with significant confidence, overall, for individual food categories. Moreover, our comparison to the UK sugar reduction program meant we were able to provide a real-world recommendation about how the Australian program could be strengthened immediately in a realistic and feasible way.

Limitations to the analyses are that households report the amount of purchased foods and beverages, not the amounts consumed, and wastage is therefore not incorporated into the estimates. It is also likely that there is under-reporting of purchases which has been previously estimated to be about 10-20% for NielsenIQ Homescan data, a level that is similar to several dietary intake assessment methods [47, 48]. Moreover, NielsenIQ Homescan only collects information about food and beverages brought into the home. As we were unable to estimate the amount of sugar reduction from purchases of foods and beverages consumed outside of home such as from restaurants and cafes, it is likely we have slightly underestimated the impact of sugar reformulation targets on total sugar purchases. While the majority of main household shoppers in NielsenIQ Homescan were above 40 years of age, this is not representative of the entire sample given the main household shopper is likely to be an adult and/or parent who purchases groceries on behalf of the household. It is also likely that some selection bias is present in the NielsenIQ data given participation in the panel relies on households to volunteer. However, the use of sample weights reduced the likelihood of such sampling biases impacting the representativeness of the results. Lastly, we acknowledge that achieving maximal reductions with Australia’s sugar targets and the UK sugar reduction program is optimistic and that in reality, voluntary compliance means actual impact on population sugar intake is likely to be even lower than our estimates. Future research is needed to explore where the Australian sugar targets should be set to ensure only one-third of products meet the targets at baseline and what impact this would have on reducing sugar consumption across the population.

## Conclusion

Sugar-reduction policies, including reformulation, have significant potential to reduce population sugar intake but only if they are well designed and implemented. With a large disease burden attributable to excess sugar consumption in Australia, these data indicate an urgent need for the Australian government to review and enhance its approach to this important health challenge. Our findings suggest that the current Australian government reformulation targets will have a negligible impact on sugar consumption in Australia, because the targets set are insufficiently ambitious, and the breadth of products covered is limited.

## Supplementary Information

**Additional file 1: Supplementary Table 1.** List of the Australian sugar reformulation targets [1]. **Supplementary Table 2**. List of the food categories included in the UK sugar reduction targets [2]. **Supplementary Figure 1.** Participant flow diagram. **Supplementary Table 3.** Household characteristics of the NielsenIQ Homescan Consumer panel in 2018. **Supplementary Table 4.** Modelled impact of the UK sugar reduction targets on changes to sugar purchases (g/d per capita), by income level

## Data Availability

The data that support the findings of this study are available from NielsenIQ and FoodSwitch, but restrictions apply to the availability of these data, which were used under license for the current study, and so are not publicly available.

## Abbreviations

WHO: World health organization
NIP: Nutritional information panel
OECD: Organisation for Economic Co-operation and Development
