# Supplementary material for: Estimating the potential impact of the Australian government’s reformulation targets on household sugar purchases
Source: Int J Behav Nutr Phys Act. 2021 Oct 28;18:138. doi: 10.1186/s12966-021-01208-6 (PMC8555094; doi:10.1186/s12966-021-01208-6)
Supplement: Supplementary file 1 — Additional file 1: Supplementary Table 1. List of the Australian sugar reformulation targets [1]. Supplementary Table 2. List of the food categories included in the UK sugar reduction targets [2]. Supplementary Figure 1. Participant flow diagram. Supplementary Table 3. Household characteristics of the NielsenIQ Homescan Consumer panel in 2018. Supplementary Table 4. Modelled impact of the UK sugar reduction targets on changes to sugar purchases (g/d per capita), by income level [file 12966_2021_1208_MOESM1_ESM.docx]

| **Supplementary Table 1. List of the Australian sugar reformulation targets [1]** | | | |
| --- | --- | --- | --- |
| **Food category** | **Sub-category** | **Category description** | **Target (g/100g or 100mL of total sugar)** |
| **Breakfast cereals** | Breakfast cereals with fruit | Commercial breakfast cereals with added dried fruit. | 22.5g/100g AND at least a 20% reduction for products containing over 28g sugar/100g |
|  | Breakfast cereals without fruit | Commercial breakfast cereals without dried fruit. May contain fruit pastes/purees. Products may contain coconut. | 20g/100g AND at least a 20% reduction for products containing over 25g sugar/100g |
| **Flavoured milk** | Flavoured milk: Mammalian | Mammalian milk with added flavour(s). | 9g/100ml |
|  | Flavoured milk: Dairy alternatives | Any dairy milk substitute with added flavour(s). | 5g/100ml |
| **Muesli and snack bars** | Muesli and snack bars | Baked or cold-formed cereal-based snack bars, based on cereals and/or nuts and/or seeds and/or fruit, may contain fruit, nuts, seeds, chocolate or yoghurt chips/ or coating or other fillings and toppings. | 25g/100g AND at least a 15% reduction for products containing over 28.5g sugar/100g |
| **Non-alcoholic Beverages** | Flavoured water, flavoured mineral water, soda water and iced tea | Ready-to-drink, non-dairy beverages with caloric sweeteners, excluding soft drinks, fruit drinks and energy drinks. | 5g/100mL |
|  | Carbonated soft drinks and energy drinks | Ready-to-drink, non-dairy, sweetened drinks marketed as soft drinks or energy drinks. | A 10% reduction for products containing more than 10g sugar/100mL |
|  | Fruit drinks | Ready-to-drink fruit drink (carbonated or still), containing less than 96% fruit juice, with added sugar. | 9.5g/100ml |
| **Sweetened yoghurt** | Sweetened yoghurt: Mammalian | Sweetened dairy-based yoghurts, liquid or semi-solid. | 12.5g/100g |

1. Healthy Food Partnership. Partnership Reformulation Program – Summary of food categories and reformulation targets. Available online: https://www.health.gov.au/resources/publications/partnership-reformulation-program-summary-of-food-categories-and-reformulation-targets. Accessed on 03/09/2021.

| **Supplementary Table 2. List of the food categories included in the UK sugar reduction targets [2]** | | | |
| --- | --- | --- | --- |
| **Food category** | **Mechanism of most relevance to category** | | |
|  | **Reformulation** | **Portion size** | **Shift in product sales toward lower sugar foods** |
| Breakfast cereals | Yes |  | Yes |
| Biscuits/cookies | Yes | Yes | Yes |
| Cakes | Yes | Yes |  |
| Chocolate confectionery |  | Yes |  |
| Ice cream, lollies and sorbets | Yes | Yes |  |
| Morning goods | Yes | Yes |  |
| Puddings | Yes | Yes |  |
| Sweet confectionery |  | Yes |  |
| Yoghurts | Yes | Yes | Yes |

[2] Public Health England, *Sugar Reduction: Achieving the 20%. A technical report outlining progress to date, guidelines for industry, 2015 baseline levels in key foods and next steps*. 2017, Public Health England: London, UK.

**Households in the Nielsen Homescan panel
 (*n* = 11,056)**

Households excluded for not meeting eligibility criteria (n=3,067):

- Not on the panel for the entire 52-week period;
- Did not report purchase data for at least 50% of the weeks;
- Missing demographic information;
- Not meeting Nielsen thresholds for expenditure (≥$5 a week on all purchases).

**Households meeting eligibility criteria provided by Nielsen
(*n* = 7,989)**

Households with lowest annual food and beverage expenditure excluded (n=801):

- Households in the lowest 2.5^th^ percentile defined separately for single-member households and multi-member households.

**Households included in the
final analyses
(*n* = 7,188)**

**Supplementary Figure 1.** Participant flow diagram

**Supplementary Table 3. Household characteristics of the NielsenIQ Homescan Consumer panel in 2018**

|  |  | **Number of households (%)** | |
| --- | --- | --- | --- |
| **Characteristics** | **Classification** | **Households in the NielsenIQ panel (n=10,052)^1^** | **Households included in the current analysis (n=7,188)** |
| **Household size** | 1  2  3  4  5+ | 2215 (22.0)  3415 (34.0)  1708 (17.0)  1667 (16.6)  1047 (10.4) | 1579 (22.0)  2499 (34.8)  1230 (17.1)  1188 (16.5)  692 (9.6) |
| **Annual equivalised household income** | Low <$28,667  Middle: $28,846 - $52,778  High: >$53,125 | 3457 (34.4)  3398 (33.8)  3197 (31.8) | 2499 (34.8)  2503 (34.8)  2186 (30.4) |
| **Lifestage^2^** | Adult households  Young families  Mixed families  Older families  Older singles and couples | 484 (4.8)  2025 (20.2)  1470 (14.6)  3733 (37.1)  2340 (23.3) | 149 (2.1)  1266 (17.6)  1100 (15.3)  2977 (41.4)  1696 (23.6) |
| **Sex of main household shopper** | Males  Females | 3061 (30.5)  6991 (69.6) | 2271 (31.6)  4917 (68.4) |
| **Age of main household shopper** | Under 25 years  25-39 years  40-65 years  Over 65 years | 72 (0.7)  1803 (17.9)  5761 (57.3)  2416 (24.0) | 13 (0.2)  823 (11.5)  4345 (60.5)  2007 (27.9) |
| **Education level of main household shopper** | Less than high school  Highschool/trade/diploma  Bachelor’s degree or higher  Not stated or other | 1957 (19.5)  4066 (40.5)  2440 (24.3)  1589 (15.8) | 1476 (20.5)  2985 (41.5)  1602 (22.3)  1125 (15.7) |

^1^The NielsenIQ Homescan Panel comprised 11,056 households, of which 10,052 reported demographic information. ^2^Lifestage classifications - Adult households: All persons aged≥18 year old, excludes all other age groups; Young families: adult shoppers any age, children <11 years old; Mixed families: adult shoppers any age, ≥ 1 children <11 years old, ≥1 children 11–17 years old; Older families: adult shoppers any age, children aged 11–17 years old; Older singles and couples: all adults > 45 years old, no children, 1 or 2-person households.

| **Supplementary Table 4. Modelled impact of the UK sugar reduction targets on changes to sugar purchases (g/d per capita), by income level** | | | |
| --- | --- | --- | --- |
|  | **Mean sugar purchases across targeted food categories**  **(g/d per capita)^2,3^** | | |
| **Income level^1^** | **Current** | **Sugar levels reduced by 20%** | **Difference (Current – targets applied)** |
| Low | 24.5 | 19.8 | 4.8 |
| Middle | 20.0 | 16.1 | 3.9 |
| High | 18.8 | 15.2 | 3.7 |

^1^The OECD-modified equivalence scale was applied to calculate equivalised household income (adjusting for household size and age of household members). Three income groups (low, middle and high) were then generated by splitting households into three groups of approximately equal numbers. The three household income groups (low: <$28,750 per year, middle: $28,750–55,000 per year and high: >$55,000 per year) had mean incomes that were comparable to equivalised incomes for the Australian population in 2016 (low income: ≤30th percentile ≤$33,020; middle-income: 30–60th percentile: $33,021–51,324, high-income: >60th percentile ≥$51,325).^2^SE for sugar purchases (g/day per capita) not displayed as SE ≤0.01 for each mean value. ^3^Results are sales weighted and are projected to the Australian population using sample weights provided by NielsenIQ.
